# The bounds of suicide talk: Implications for qualitative suicide research

**DOI:** 10.1177/13634593211060767

**Published:** 2021-11-27

**Authors:** Patti Ranahan, Veronica Keefe

**Affiliations:** Concordia University, Canada

**Keywords:** focused ethnography, rural health, suicide prevention

## Abstract

Following the implementation of a provincial suicide prevention gatekeeper training initiative in western Canada between 2015 and 2018, we conducted a focused ethnography designed to capture the post-initiative context within one small community. Analyses of our field observations and interviews with community members suggest suicide prevention work is represented in multiple informal or coordinated actions to generate innovative pathways to provoke open conversations about suicide. Simultaneously, suicide talk is constrained and managed to limit vulnerability and exposure and adhere to community privacy norms. Further, parameters around suicide talk may be employed in efforts to construct the community and mental health care in livable ways. As the research process paralleled existing representations of suicide prevention work in the community, this paper explores our entanglement in the bounds of suicide talk during phases of recruitment, data collection and knowledge translation activities.

Suicide, and its complexities, are rendered visible by how discourse is controlled or freely expressed. Parameters are established around suicide talk in multiple ways. For example, guidelines are offered in sharinglived experiences (e.g. Suicide Prevention Resource Center, 2020), and trigger warnings may be used to notify forthcoming discussions of difficult or sensitive topics, such as suicide, that may provoke strong emotions (Boysen et al., 2016). Communicated verbally (Boysen et al., 2016), and with the aim of creating a “safe space” for discussion (Carter, 2015: 11), trigger warnings establish a boundary around suicide talk. Also evident in guidelines for media reporting on suicides are efforts to delimit suicide talk so it is not harmful (Duncan and Luce, 2020). Indeed, there is a demonstrated relationship between media reports of celebrity deaths by suicide and publication of methods with increases in suicide risk in the general population (Niederkrotenthaler et al., 2020). Popular shows focused on the topic of suicide (i.e. *13 Reasons Why*), are associated with notable increases of suicides among young people (Bridge et al., 2020; Niederkrotenthaler et al., 2019). Constrained suicide talk can extend to the client-therapist relationship as many clients actively conceal or self-censor suicidal thoughts in psychotherapy (Blanchard and Farber, 2020). For the purposes of our discussion, we refer to suicide talk that is limited, constrained or procedural as *bounded* suicide talk.

Notably, efforts to bind suicide talk is situated in stark contrast to suicide prevention messaging and intervention practices. Promoting open discussion about suicide is often a key message in suicide prevention efforts (Ftanou et al., 2017), and not all suicide talk leads to adverse outcomes. For example, viewing the series *13 Reasons Why* generated interest in some young adults in providing help to a person thinking of suicide (Arendt et al., 2019), and for parents and teens, provided opportunities to connect and process challenging topics such as suicide, depression and bullying (Lauricella et al. (2018). McGorry (2011: para.11) warns of youth being “trapped in a bubble: a cone of silence” in the absence of being offered permission to talk about feelings of suicide, while Fitzpatrick and Kerridge (2013: 471) suggest that “trust in people’s capacity to reflect on even the most difficult issues” must replace the need to control suicide talk. Suicide intervention practices often involve sharing disclosures widely, including communicating with professionals or service providers unknown to the client (Ranahan, 2013). Navigating the bounds of suicide talk is a thorny issue that holds implications for conducting suicide research especially within small communities “where strangers are few and personal information seems to belong to everyone” (Wilson-Forsberg and Easley, 2012: 281).

In this paper, we consider the implications of the bounds of suicide talk that arose while engaged in a focused ethnography within a small rural community. Our research journey mirrored our participants’ stories and our field observations. First, we describe the methodology, situate the study, and outline the research procedures. Next, we present findings from our analysis, which suggest significant efforts are carried out in multiple informal and coordinated actions to generate innovative pathways to igniting and broadening conversations about suicide. Simultaneously, some community members are actively working to restrict talk of suicide, rendering some conversations impeded, and others, restricted or requiring adherence to a particular narrative. Lastly, we integrate relevant literature in our discussion of the implications for conducting qualitative suicide research within small communities that arose during our study.

## Methodology

To examine our research questions, we conducted a focused ethnography endeavoring to capture the contextual factors implicated in local suicide prevention efforts within one small community (White, 2016). In this section, we describe the provincial suicide prevention gatekeeper training initiative that preceded the present study. We identify our research questions, our rationale for the methodological approach, and our procedures.

### Background context

Prior to our study, rising suicide rates in British Columbia, Canada, led to a 3-year province-wide initiative to implement suicide prevention gatekeeper training between 2015 and 2018. Gatekeeper training programs are designed as brief workshops that aim to increase awareness about suicide, recognition of indicators of distress, responsiveness in conversations with persons contemplating suicide, and referrals to supports. Gatekeeper training is a promising effort when implemented as part of a larger suicide prevention strategy (Griesbach et al., 2008; Walrath et al., 2015). With the aim of having 20,000 citizens trained in either Applied Suicide Intervention Skills Training or SafeTALK (LivingWorks, Inc, n.d.), various stakeholders contributed to implementation efforts. While conducting a focused ethnography on the implementation of the initiative (Ranahan and White, 2019; White and Ranahan, 2020), a movement away from the initiative’s original goals was observed. This movement was directed toward generating pathways to helping, constructing responsibility to help, and negotiating communities of place. Findings indicated that various communities throughout the province held different degrees of readiness to embrace participation in training. In some communities, grieving and healing from suicide bereavement were understood as prerequisites to learning about suicide intervention skills and helping others in distress. Further, the natural and built environment intertwined with the social context of implementation. For example, training required in-person delivery, however not all communities were accessible by road during the winter months. Rural communities were responsive to their town’s unique values and norms, such as expectations for in-person promotion of the training. The initiative transformed during implementation as new collaborations, relationships, and activities took hold in ways that mattered to within local communities across the province.

After the conclusion of the gatekeeper training initiative, Ranahan returned to one rural British Columbia community between October 2019 and February 2020 to conduct a focused ethnography exploring the post-initiative local context, with Keefe as a Research Assistant on the project. This study was guided by the following research questions: (1) What were the intended and unintended effects of the gatekeeper training initiative within a local rural community? and (2) How do community members represent their understanding of life promotion, suicide prevention, and livable futures in concepts, beliefs and values embedded in local practices?

Focused ethnography was well-suited for exploration of the corollaries of the gatekeeper training initiative within a local context, and how community members represent their understandings of life promotion, suicide prevention and livable futures within local practices. Focused ethnography is a promising method for exploring a distinct issue, such as suicide prevention work, within a specific setting, to understand the complexities around the issue from the participants’ perspectives (Cruz and Higginbottom, 2013). Focused ethnographies have a clear purpose and are characterized by a focus on a discrete community, a focus on a specific problem or social phenomena, episodic participant observation, and involvement of a limited number of participants who have specific knowledge about the phenomena (Muecke, 1994). With its origins in community-oriented health sciences, focused ethnographies are exploratory and time-limited, drawing upon data from brief field observations at selected events and times, and unstructured and semi-structured interviews with key informants (Muecke, 1994). As such, focused ethnographies are data intensive, gathering a large amount of data within a short period of time (Knoblauch, 2005). While traditional ethnographic approaches position the researcher as objective observer, researchers utilizing focused ethnography are positioned as insiders holding sufficient background knowledge and experience with the field of study (Wall, 2015). Knoblauch’s (2005) recommends that the researcher has prior intimate knowledge of the field to be studied. Ranahan had previously established research relationships in the community vis-à-vis the provincial gatekeeper training initiative. Ranahan had also lived and worked in a neighboring community and was familiar with health and social service structures and geographic landscapes of the region.

### The study setting

In discussing ethnographic approaches, Muecke (1994: 203) states: “What is most important is that the people studied be contextualized comprehensively and accurately in their local symbolic, social, and physical environments.” The discrete community that served as the study setting is located in the southern part of the western Canadian province of British Columbia. This setting was chosen as previously established relationships were in place with community stakeholders charged with implementing the gatekeeper training initiative. The community was recognized for its novel suicide prevention efforts, including an established suicide prevention committee, an annual suicide prevention awareness event, and new investments in integrated youth mental health services.

Geographically, the natural landscapes of mountains, lakes, valleys, wooded areas and navigable shorelines wrap the community. Rooted in farming and agriculture, forestry, manufacturing, machinery and tourism, the largest town has a population of almost 18,000 citizens. Relative to the provincial average, community members have lower household incomes, more trade and college graduates than university graduates, and a slightly lower unemployment rate (Provincial Health Services Authority, 2020; Statistics Canada, 2016). Housing affordability is a concern, with nearly half of renter households spending more than 30% of their total income on rented property. Being a rural community, the primary mode of transportation is by car with less that 2% of employed adults take public transportation (Provincial Health Services Authority, 2020; Statistics Canada, 2016). The majority of residents report very good or excellent mental health, have a strong sense of community belonging, and have a life expectancy on par with the provincial average (Provincial Health Services Authority, 2020). This health region holds the highest rate of suicide deaths in the province at 16 deaths per 100,000 (BC Coroners Service, 2020). The provincial suicide rate in 2018 was 12 deaths per 100,000, slightly higher than the national rate of 10.3 deaths per 100,000 for the same year (Centre for Suicide Prevention, 2020). The community exceeds the average for access to physicians, but falls far below the provincial average for access to health care specialists (Provincial Health Services Authority, 2020). As police often serve as the initial point of contact in rural and remote communities (Vaughan, 2017), and are responding more to incidents involving mental health (Shore and Lavoie, 2019), provincially, smaller communities have a higher rate of police-involved deaths (BC Coroners Service, 2017). Health and social services within the region are largely divided between publicly funded services and non-profit organizations, who receive funding from a variety of sources including provincial government contracts, donations, grants, and services.

### Procedures

Institutional ethics approval was obtained in September, 2019. In collaboration with a community non-profit organization, third-party recruitment efforts, purposive and snowball sampling strategies generated adult volunteers representing diverse groups within the community. Respondents were situated within the community as service providers, volunteers within social service organizations, citizens, health care providers, school personnel, municipal workers, and persons with first-hand lived experience (e.g. service users with previous suicide attempts and/or experiences of mental illness and suicide ideations, persons who lost a loved one to suicide). Data were gathered between October 2019 and February 2020, and were comprised of individual audio-recorded semi-structured interviews (*n* = 23), 7 hours of selected field observations, researchers’ notes and memos, and relevant documents. Interviews were 60 to 90 minutes in length and conducted in-person or on the phone at a time and place convenient for the participant. Keefe transcribed the recordings verbatim. The written consent/assent process was facilitated with each participant prior to engaging in interviews and field observations. Field observations were conducted at selected events (i.e. a suicide prevention coalition meeting; *n* = 7) and specific settings (e.g. integrated youth health center; *n* = 7). Two-hours of field observations were conducted in public settings identified by participants as spaces that promoted wellbeing (e.g. grocery store). Documents offered by participants (e.g. poster advertising speaker on mental health, brochures, etc.), artifacts (e.g. photographs), and community health data (e.g. Canadian Community Health Survey 2015–2016), were gathered for inclusion in the analysis.

### Data analysis

Analysis was informed by a relational constructionist framework, with a focus on relational processes and the ways these processes work together to construct beliefs, values and concepts of suicide prevention, livable futures and life promotion in the post-initiative community context (McNamee and Hosking, 2012). Relational realities are co-constructed through language and the ways of human relating through language (Hosking and Pluut, 2010). The analytical process was iterative, yet structured, with multiple readings of transcriptions, field notes and documents, and engaging in weekly discussions. Transcripts were initially open coded (Corbin and Strauss, 2014) and emerging themes were documented in an expansive table, that was revised as data was revisited and triangulated. Data organization and management was initially organized chronologically, and as the study progressed, categorized and re-categorized by themes, and visual renderings of connections between data (Coffey, 2018). In the next section, we describe themes identified during our analytical process.

## Findings

Analyses of the data led to the identification of two overarching themes: (1) generating pathways to conversations about suicide, and (2) bounded suicide talk. Notably, the two themes are dialectical, with conversations being opened up, while concurrently being closed down or restrained. Here, we offer a detailed description of each theme, drawing upon participants’ quotations for illustration purposes.

### Theme 1: Generating pathways to conversations

Generating pathways to begin conversations about suicide are at the forefront of life promotion and suicide prevention work within the community. These efforts are focused on broadening the conversation around suicide, ensuring visibility of the work, and promoting educational activities and awareness in multiple formats and spaces. Within these spaces, conversations require provocation and exposure. Participants’ lived experiences and training opportunities in suicide prevention were conditions that provoked and exposed community members to conversations. Training, in particular, was an identifiable space that rendered suicide talk permissible, yet procedural. Completion of training denoted a level of expertise, confidence, comfort and openness to engaging in conversations about suicide. In the following paragraphs, participants’ quotations illustrate the generation of pathways to conversations about suicide.

Stories and conversations about suicide can remain hidden unless a pathway is provoked. Participants described suicide prevention work in the community being driven by outreach efforts to create space for conversations about suicide to occur. Participant 2, a mental health service provider, describes creating opportunities for dialog:

We went to different places in the community to do that outreach piece and really found that people have stories to share [. . .] there are folks out there who want to talk and who want to reach out. It’s just a matter of making finding that right opportunity for them.

Increasing exposure to suicide prevention information is viewed as desirable. Participant 1, a mental health service provider, suggests, “the more exposure [to the topic of suicide], the better.” Open and direct conversations were strategies to combat stigma within the community. Participant 5, a service provider and person with lived experience, explains: “Just sending the word out there has been helpful because then people can talk about suicide openly and directly without feeling the shame and stigma.” “Send the word out” within the community increases exposure to information about suicide prevention. Information is spread by posters and community calendars on bulletin boards available in community centers, drop-in spaces in non-profit organizations, and in bathroom stalls. Participant 5 explains, “When you go in the bathroom, you’ll find a sign or a poster (about life promotion programs) there.” Indeed, during field observations, an artifact (i.e. mason jar lantern) from a prior suicide awareness event in the community was present in a restaurant bathroom, conspicuously positioned on the counter with the message “Dream.” Mason jar lanterns were associated in the community with an annual suicide prevention awareness event in which a lake in the center of town was lined with lanterns. Participant 11, a volunteer at an organization and person with lived experience, describes the need for prolific information, even if community members were not necessarily looking for, or desiring, it: “Just everywhere, that you could access it like instantly, even if you didn’t want to access it, you would walk past it.” Being immersed and exposed to information and provoking suicide talk underpinned suicide prevention work in the community.

Igniting one conversation, catalyzes further talk of suicide within the community. Sharing lived experiences with mental illness and suicide provokes further conversations. Participant 3, a person with lived experience, explains, “I had a mental health illness and in sharing that, people began to talk about it. . . it just opened up the wider conversation about mental illness and suicide.” With greater frequency and exposure, Participant 6, a volunteer and person with lived experience, describes a shift in the community toward normalizing suicide prevention conversations: “I think just like hearing so much of that around me, kind of normalized it a bit more. It’s like, this is just a conversation we can have. It’s just like any other conversation.” When conversations are ignited and new pathways for dialog constructed, sharing is experienced as rewarding. Participant 22, a person with lived experience, explains, “It was very beneficial for me to try and understand that I wasn’t alone too. And, there were other people that were having issues.” Participant 11 echoes feeling less isolated upon hearing about others’ experiences with suicide: “It’s just really nice to know that you’re not the only person in the boat,” utilizing a metaphor used to describe being in the same situation as other people. Conversation with others reveals the shared experience of suicide between community members, and challenges perceptions of suicide being an individual, singular experience.

Some participants report their involvement with suicide prevention work, volunteer or occupational, as meaningful and motivating. Participant 9, a volunteer and person with lived experience, explains, “It just makes me feel like I have a function or a role,” and Participant 13, an advocate and person with lived experience, states, “I knew that if I just got out there [to talk about suicide], I could impact a lot of people. So that’s one thing that helps drive me.” Further, Participant 14, a municipal worker and person with lived experience, identifies engaging in suicide prevention work as “personally meaningful,” and Participant 2, a mental health educator, espouses the belief that “we all have something to give.” Suicide prevention work can be viewed as meaningful and rewarding when community members can envision ways to contribute to information spread.

Suicide prevention training is a pathway to a permissible space for conversations about suicide. At times, training is identified as a space to seek help for suicide ideation, as Participant 2 explains: “[A participant in training] had actually chosen to come to this course because they were thinking about suicide and intent on suicide.” Training is advertised and often open to anyone in the community to participate. Participant 14 identifies training as “the beginning of the conversation.” Suicide is a topic deemed uncomfortable or awkward, therefore training is a way to mitigate the fear of engaging in conversations by offering a structure around how to talk about it. Participant 8, a volunteer and person with lived experience suggests, “I really think we need to offer safeTALK so that people will get a little more friendly with the word [suicide].” Building confidence and comfort when discussing the topic of suicide is attributed to engaging in training as Participant 6, a volunteer and person with lived experience, explains: “Taking safeTALK and then I took ASIST too, the 2-day training. . . Like those definitely helped me be more comfortable talking.” Participant 10, a person with lived experience, explains further how training provided guidance on how to talk about mental health and suicide, which built their confidence:

It gives me confidence to talk to them about it, and talk differently about it. Because I probably, well I don’t know what I would have said before. Now I have a few words and a little bit of knowledge. The biggest thing is just giving me confidence to be able to have a conversation.

Training as a pathway to knowing how to talk about suicide and engage in conversation is discussed by Participant 5 as they reflect on a friend who was struggling:

I had a friend. . . She just says, “I’m going to end it all.” And so there I was like, “I don’t know what to do. Like, I’m not trained to help you.” . . .The support wasn’t like I would support them now with the knowledge that I have [after completing gatekeeper training].

Training is valued and viewed as propelling individuals, and the community, forward in suicide prevention work, with some participants expressing gratitude for having the opportunity to take part in the preceding provincial gatekeeper initiative, because “If we didn’t have that training or like that that person to spearhead the whole thing then [the community] would not be where they are today” (Participant 8). Knowledge from training repositions suicide from a private experience to a known experience. Participant 3 explains how they suggested to others that training was helpful in delineating how to talk to people about suicide so it does not have to be a “secret”:

I said, “You know, [gatekeeper training programs] are really good because they give you some tools. How to talk to people.” . . . I showed them the little cards that I got at the course. . . I can sit beside someone and show it to them. So, it’s not a secret. It doesn’t have to be a secret.

Suicide prevention work in the community centers on generating pathways to conversations. These conversations are ignited by creating space for dialog, increasing the spread and exposure to information about suicide, sharing lived experiences, and participating in training.

### Theme 2: Bounded suicide talk

Despite prolific efforts to initiate conversations about mental health promotion and suicide prevention, simultaneously dialog is constrained and managed. Suicide talk is bounded by fear of others’ responses, a desire for unentangled spaces free from a focus on suicide or mental health, or constrained by community privacy norms, stigma and procedural interactions. Participants’ stories revealed that there are acceptable, and unacceptable, safe and unsafe, ways of talking about mental health and suicide. There are accepted, and unacceptable, spaces, times and persons to have conversations with. When the ways of talking about suicide and mental health are not deemed acceptable, talk can be suppressed or restricted.

Binding suicide talk may be an effort to exercise autonomy and control over one’s life. The purposeful omission of information works to control the anticipated responses of others. For example, opportunities to explore mental health and suicide may be constrained even when accessing help. When medications are offered as a remedy, some participants experienced this as a way of closing down any talk about suicide or mental health and not addressing the participant’s core concern. Participant 7, a person with lived experience, explains: “I got sick of the medications. They kept adding medication upon medication upon medication, so that’s not helpful. To me, it’s like you fall into a dark hole and medication is like furniture for your dark hole.” Participant 7 shared they subsequently weaned themselves off medication without initially informing their doctor: “I did that on my own to begin with because I don’t think it would’ve been really supported.” Fears about how the provision of care will unfold, the lack of support and the potential loss of control of rights and autonomy constrains talk of suicide. Service users learn the rules of engagement (e.g., what to disclose, how the system will respond) and the bounds of suicide talk in interactions with health care providers. Participant 18, a person with lived experience, explains learning how to limit what they shared and “keep quiet”:

The [hospital] staff could obviously. . . knew [about feeling suicidal], you know? They would say to me “If we leave you alone, you’re not going to jump out the window, are you?” Yeah, but on the one hand, so that was good. But on the other hand, I think maybe it taught me to keep quiet, which isn’t necessarily a good thing.

Participant 18, further describes constraining talk of suicide when calling the distress line for mental health help: “When I phone the crisis line, it’s knowing what not to say so that they don’t phone 9-1-1.” A health care provider, Participant 20, described working with a patient who sought help for mental health and suicide ideation from a psychiatrist in the community:

I had a patient who went to [the psychiatrist] once and said, “I’d like to talk some more about my problems.” And [the psychiatrist] said “I’m not here to talk to you. I’m here to prescribe medication.” And I remember hearing that and my jaw hit the floor.

Service users also learn to restrict suicide talk in screening or intake forms. For example, when completing questionnaires or assessment forms, service users may not offer up authentic responses. Participant 11 shared, “It’s a 10-question questionnaire. . . I don’t really have time for a mental episode right now, so I’m just going to say ‘no’.” Bounded suicide talk is evident in participants’ limiting disclosure of suicide ideations while completing questionnaires, or not mentioning “suicide” during a call to the crisis line, and sharing how they learned to constrain suicide talk when accessing help.

At times, finding spaces without the expectation of having to talk about one’s mental health or suicidal ideations is beneficial. Participant 18 explains:

Get in the car and drive to the grocery store, and just be there where other people are, and focus on getting groceries and not having to carry on any lengthy conversations with anybody, which would be very difficult then. But it usually really turns me around. There’s a couple of there’s a women’s clothing stores downtown. I know the people who work there and I can go and they’re just cheerful and “Anytime, come anytime you want. You know, you don’t have to buy anything.” They don’t realize how low I am because I don’t speak to people about that.

Finding spaces that are free from suicide talk are ways that some community members are instituting parameters the spread and discussion of suicide. In this way, bounded suicide talk allows participants to construct their community in ways that are livable.

Suicide talk can be controlled due to community norms about privacy thus limiting what information is circulated. Participant 6 explains the uncertainty of the information available about a death by suicide in the community:

I don’t always know if the full story. . . Just last week someone died by suicide there. So, from my understanding that was a lack of supports and maybe even just like awareness and stuff there. But I don’t know the full story.

While privacy norms may limit suicide talk, strategic information about suicide is purposefully shared. For example, statistics are used purposefully to “convince” people of the importance of the topic. Participant 4, a mental health service provider, is “keeping track” of the number of situations involving suicide in the organization to ensure the sustainability and value of the service and “to use that data to convince people.” Further, training suggests ways to strategically and procedurally engage in conversations about suicide. For example, Participant 5 explains listening to someone with the purpose of finding a “turning point”:

It was kind of practical for me to kind of walk through those steps and figure out, do they have a plan? They told me that they had a plan. They’re going to kill themselves in 2 months. And so just, kind of listening to their story and trying to find a turning point for them in their life.

Suicide talk is also bound by the expectation of specific knowledge and expertise and occurring with identifiable safe persons at specific times. Participant 3, a service user and person with lived experience, explains having training in suicide prevention provides knowledge for how to talk to people: “I heard about the [gatekeeper training] programs. I decided that I wanted more knowledge so that when I talk to people. . . I wasn’t just winging it on my own.” Bounded suicide talk unfolds at particular times and in specific ways with identifiable “safe” people who are knowledgeable and trained. For example, Participant 5 identifies themselves to others in the community as a “safe person” to talk to: “Wherever I go. . . I let them know ‘I’m trained in suicide prevention. If you want to talk about it, feel free to talk about it. I’m a safe person to talk to.’” Participant 12, a mental health service provider, explains the bounds of the therapeutic process describing how talk unfolds at particular times:

Once a week, [at] this time, you go ahead and talk about your problems, right? You open up the box of problems, and then you close it afterwards. . . I think it’s actually really healthy thing to be holding back your problems for a time and then finding the right places, at the right times, to be letting them out.

Controlling information can be a strategy employed to protect oneself or others, with respect to privacy, confidentiality, and/or exposure/vulnerability. Participant 4 explains the protection of information in how mental health appointments are recorded: “Even with the appointment card, it doesn’t say why, just that they were in, because we’re protecting their confidentiality, right?” Information about mental health and suicide is positioned as private, requiring protection and having the potential to harm.

Boundless suicide talk can lead to feelings of embarrassment and exposure, with significant consequences. Participant 3 identifies the fear and embarrassment associated with disclosing mental health issues: “Because people are afraid to talk. They’re embarrassed.” Participant 7 suggests disclosure is exposure: “I haven’t really put myself out to go to get help. It’s kind of a vicious cycle. You have to get motivated to get up and reach out and then expose yourself and have to follow through.” Disclosure of mental health issues can lead to negative outcomes. For example, a disclosure to their employer about difficulties with depression led to a decrease in hours and eventual loss of employment. Participant 7 explains: “I lost my job because of it. Yeah, so you feel like the mental health thing, once it’s out, it’s out of the bag.” Stigma renders community members silent. Participant 14 explains: “They are struggling with mental health silently because you can’t talk about that to any of your buddies. Or you know, seeking help is going to stigmatize you.” Fears about offending others if asking about suicide also invites silence with the potential for significant consequences. Participant 5 describes the challenge of community norms around privacy: “Here, it is like, I don’t really want to ask too much. Like I don’t want to offend them. But in the process of doing that, we lose people [to suicide].” Suicide talk is constrained by community norms about privacy, stigma, and desires for autonomy and spaces free from a focus on suicide and mental health problems. Identifiable safe persons who are knowledgeable and trained are positioned as those who can readily engage in suicide talk. Parameters around suicide talk are also rendered visible in interactions with service providers where participants learn what is acceptable talk, and how to avoid unwanted responses.

Underpinning efforts to generate conversations is the assumption that every space, is a good space for discussions about mental health and suicide. This assumption catalyzes the re-imagination of existing spaces and roles as places and connections where suicide prevention work can occur. Indeed, churches, bible study groups, mom support groups, hair salons, the farmer’s market, arts and sewing clubs, on social media, the grocery store, the thrift store, or participation on sports teams, are cited by participants as places within the community where conversations can, and do, occur. Simultaneously, suicide talk is bounded by community norms about privacy, strategic and procedural aims in sharing information or intervening, the threat of exposure and loss of rights, and the assumptions about the therapeutic process.

## Implications for qualitative suicide research

In this final section, we offer a reflexive account on the implications for conducting suicide research in light of our research process, findings, and relevant literature. As Dickson-Swift (2019: 3) explains, “undertaking qualitative research is often an emotional journey, not only for the participants but for others that may be involved along the way.” Indeed, our research journey was marked by tensions when translating our findings as we were caught up in the landscape of bounded suicide talk. First, we discuss how the data collection process unfolded in existing representations of suicide prevention work in generating pathways for conversations, while simultaneously binding suicide talk. Second, we consider our entanglement in the bounds of suicide talk when aiming to share information about the research project and our findings.

### Data collection and bounded suicide talk

Igniting conversations and creating pathways for discussions about suicide is aligned with broader calls in the literature for more public awareness campaigns as part of suicide prevention efforts (Fitzpatrick and Kerridge, 2013). Suicide prevention efforts within the community were represented by generating pathways to conversations and opening doors for suicide talk in novel spaces. Identifiable ways to connect, to build relationships, to offer a listening ear, are part of low-barrier suicide prevention work in the community as evidenced in our field observations of the integrated youth mental health services “drop-in” space, the availability of a 24-hour crisis line, availability of grief and substance use support groups, and the established suicide prevention committee.

Mirroring community representations of suicide prevention work, our recruitment efforts provoked conversations. As community members responded to recruitment calls, the researcher-participant relationship constituted an additional space for suicide talk to occur. Recruitment conversations about the study revealed a yearning for a place to share lived experiences among study volunteers. Respondents described feeling “compelled” to contact us when hearing the study was about suicide. Permeating initial contacts were statements such as, “I saw this [study] was about suicide and I just had to contact you” or “I need to tell this story.”

However, the bounds of suicide talk were also evident in the research process. Notably, for those participants with lived experience of suicide, they informed us they undertook significant investments were undertaken in preparing for the research interview. Prior to interviews, participants described mobilizing existing supports and social connections to discuss what could be beneficial and challenging about engaging in the study. Some participants told us they organized post-interview counseling appointments or readied social supports (e.g. friends, family members) so they were “on hand.” Researcher-participant discussions on determining the location of the interview also indicated participants’ careful consideration of comfort and confidentiality. For example, interviews were scheduled after work hours at a participant’s place of employment once all employees had left the office.

Markedly, Ranahan also engaged in parallel preparations for the research interview. These preparations unfolded like routine procedures, from reviewing the initial emails from respondents for details of their stories, their role in the community, and demographic details, to carefully checking the recording device, reviewing the interview guide, readying the signed consent form for review with each participant, and having on hand a list of supports and services should the participant require them. Upon reflection, Ranahan’s procedural construction of the interview space undoubtedly influenced the quality of the researcher-participant relationship, potentially limiting participants’ disclosure. Social support and belonging are protective factors against psychological distress in small communities (Roy et al., 2013), yet while some helping spaces offer points of connection, not all provide a meaningful relationship. Service user participants, in particular, identified a disclosure-equals-exposure equation, that works to bound suicide talk and limit relational engagement. Evident in our findings were participants’ stories of constricted time with mental health clinicians, and how questionnaires, for example, worked to shut down discussions. Such routine procedures, while designed to be supportive and offer safety, can work to disrupt relational engagement (Ranahan, 2014), including within the researcher-participant interaction. For example, the recruitment phone conversation often unfolded into participants beginning to share their story as part of an explanation for their interest in participating. The rapport and sense of safety built in the initial recruitment call extended into the face-to-face interview as several participants continued on sharing their story where they had left off. The procedures of reviewing consent and the interview process, and turning the audio recorder on, worked to create parameters around the conversation, crafting a space to talk about suicide.

Set apart from other spaces deemed acceptable for suicide talk, the research interview crafted a unique intertwined encounter for participants and researcher alike; the fragile in-between (Boden et al., 2016). Efforts to generate pathways to conversations in community suicide prevention efforts had opened doors to spaces otherwise closed, yet according to participants, these opened spaces remained bounded. Rural and small communities are often thought of as supportive, yet a lack of confidentiality and anonymity can hinder seeking help for mental health concerns (Smith et al., 2008). Emboldened, perhaps, by the concretized commitment to confidentiality and the interest of an outsider, within the research interview space, suicide talk held the potential for unfolding in unconfined cathartic ways. Although not entirely risk free (Gibson et al., 2013), prior research suggests that participation in suicide research is experienced as positive and characterized by improvements in well-being, a desire to contribute, and the cathartic value of talking (Biddle et al., 2013; Dyregrov et al., 2011). While the construction of the research interview may well be viewed as an open, acceptable, cathartic space, of ethical concern are “whose voices are (not) included, who’s muted and who’s silenced” (Hosking and Pluut, 2010: 69). Of the 32 respondents to the initial call, two respondents did not meet inclusion criteria, and 23 committed to participating in the interview process. Foreseeably, respondents who did not participate in the study had an experience to share, yet these narratives, and the narratives of community members who did not respond to, or see, our recruitment calls, are inevitably excluded.

For those who participated, as an open and novel space for suicide talk, the research interview served as a place to foster connection. Following one particularly emotional interview, a participant shared that they viewed the researcher as an “angel” for choosing this particular community to study, and expressed a belief that perhaps, because of the study, responses to suicide in the community may change. At the close of this interview, the participant asked if they could hug the researcher. Given participants’ shared experiences regarding the personal meanings attached to engagement in suicide prevention work, participants expressed curiosity about Ranahan’s connection to the topic of suicide. Acknowledging the influence of the researchers’ positionality, especially for researchers with personal experience of the topic under study, is of the upmost importance in promoting rigor in qualitative research (Cruz and Higginbottom, 2013). Positionality locates the researcher in relation to the study subject, the participants, and the research process and context (Holmes, 2020). Demarcating such interactions between researcher, methodology, settings, and participants is required (Denzin and Lincoln, 1998). Ranahan’s personal and professional experiences with the issues being studied and prior knowledge of the context offered ease of access to the small community’s culture and understanding of colloquial language and suicide prevention discourse (Holmes, 2020). Indeed, participants may be more willing to engage in the interview if they perceive the researcher as empathic to their experiences. Having shared experiences with suicide, loss, and precarious living, Ranahan was sensitized to explore nuances, probe efficiently and compassionately (Berger, 2015). Common ground may sensitize, yet also limit the stories shared, suicide talk produced, if analysis was directed by personal experiences (Berger, 2015). Several reflexive strategies were used throughout the research process, including journaling and audio-recorded reflections, debriefing, triangulation, and participant member checking. While these strategies may well render the inquiry rigorous, our relational constructionist approach would posit that participating in the study constructs and reconstructs peoples’ lives (Hosking and Pluut, 2010), including the life of the researcher.

### Bounded knowledge translation efforts

Congruent with calls for prioritizing research on the wider societal influences on suicide and increasing the utility of findings (Niner et al., 2009), we strived to share information about the research project and our findings as they emerged. Armed with preliminary analysis and a desire that paralleled our participants’ yearning for dialog, we sought mechanisms for translating knowledge. Unknown at the outset of the study, choosing the community as a research site conveyed to the larger community audience that their stories of suicide mattered in new ways. Ranahan was asked on several occasions by community members, service providers and the media why the specific community was chosen as the research site. Our desire to share met the community’s yearning to matter, leading to multiple requests for communications about the research project.

Media communications foregrounded initial knowledge translation efforts. A brief mention in the local community newspaper led to radio interviews in neighboring towns, while a news bulletin at our university led to a TV and radio interview about the project in a large urban center 4500 km from the research site. Translating the findings in media outlets engendered similar bounds around suicide talk demarcated by our participants. On-air suicide talk was bounded, and at times procedural. For example, every interview included information on how to contact the local crisis line, a list of “warning signs” for suicide, and strategic attempts to inject hope and optimism while sharing stories of suffering, despair and precarious living.

Off-air, the lines demarcating the boundaries around suicide talk blurred unfolding as a new acceptable space for journalists to share their experiences with suicide. A nondescript off-air mention of a recent death by suicide in the community by Ranahan was transposed into a journalist’s on-air inquest with a representative from the health service provider in the region. What originally positioned community members’ stories as valuable and mattering in drawing attention from an outsider, evolved into queries on the (dis)proportionate suicide rate in the region, repositioning health care service providers on the defensive. Upon debriefing with a key expert and mentor, and in consultation with our community partner, directives on what was to be shared, and with whom, bounded suicide talk once again.

Communicating findings to participants and the community-at-large also offered up ethical challenges. Our knowledge translation strategies included participant member checking, a comprehensive report provided to the community partnering organization, and providing an infographic for local circulation. Knowledge translation includes the “synthesis, dissemination, exchange and ethically-sound application of knowledge to improve the health of [citizens], provide more effective health services and products and strengthen the health care system” (Canadian Institutes of Health Research, 2020: para.1). Member checking is a strategy used to enhance credibility of qualitative research (Creswell and Miller, 2000), while authentic reciprocity is viewed as an ethical imperative in community-engaged research (Naidu and Prose, 2018). Returning information to the community wrapped us into the existing privacy norms around suicide talk portrayed in our participants’ stories. Our initial report to the community organization was an exhaustive 16,000-word document, privileging the detailed stories of our participants, yet leaders within the organization wished instead for a list of specific actions and next steps. In response to our 1-page summary for participant member checking, several participants then requested a lengthier, more detailed report. Subsequently, we were asked by our community partner to create an infographic visually depicting our findings in an accessible form for distribution throughout the community in low-barrier spaces (e.g. farmers’ market). As we endeavored to engage in suicide talk to share our findings, we were entangled in editing for length, acceptability and circulation within multiple spaces. As Crtichely et al. (2006: 83) suggest, research relationships extend beyond the interview process and continue well after analysis, “underlining the importance of maintaining community access, trust and credibility during the life of the project.” The advent of the global Coronavirus pandemic further bounded our knowledge translation efforts as a feedback meeting with the suicide prevention committee was canceled. Aligned with Participant 6’s reflection on never knowing the “full story,” we experienced limited local avenues for sharing the full story of our findings in the community.

## Conclusion

While the call for *every space is a good space* is desirable, and normalizing conversations about suicide were sought by participants, Fitzpatrick and Kerridge (2013: 470) caution, “suicide is inscribed with deeply felt moral, religious and cultural meaning that will influence any discussion.” Suicide research in small communities is entangled with the inscribed norms and boundaries around suicide talk, rendering influences over the processes of data collection, analysis and knowledge translation. Thus, re-imagining the call as *every space is a meaningful space* is warranted. Generating pathways within the research process must include consideration of the meanings held by community members within the various spaces where suicide talk is provoked, exposed, circulated, or controlled. Generating pathways must include greater opportunities for broader discussions and expressions—beyond a focus on features of gatekeeper training (e.g. how to ask about suicide)—to enhance understanding of the complexities of this work. As Fitzpatrick and Kerridge (2013: 471) posit, “a genuine open discussion of suicide must be a wide discussion—not just a medical or public health discussion, but a social, cultural, moral, political and even religious discussion.” Fears of “saying the wrong thing,” not being pulled into another person’s distress, not feeling qualified to respond, or wishing to respect another’s privacy worked to prevent community members from offering support and relational connections to be fostered (Owens et al., 2011). Simultaneously, as focused ethnographers, a possible limitation of our suicide research is having a narrow focus on suicide prevention work within one small discrete community may have us “unknowingly exclude what is relevant” (Muecke, 1994: 203). Suicide prevention work is multi-faceted and complex, with many efforts hidden from view, or surreptitious. As suicide researchers, we must critically examine how our research process makes this work visible, the meanings held by our participants in our efforts to illuminate their experiences, and how we are caught up in multiple ways within the contested landscape of bounded suicide talk.
